# Sharkmer: repurposing PCR primers for targeted genome assembly using *in silico* PCR

**DOI:** 10.1093/bioinformatics/btag163

**Published:** 2026-04-07

**Authors:** Casey W Dunn, Samuel H Church

**Affiliations:** Department of Ecology and Evolutionary Biology, Yale University, New Haven, CT 06511, United States; Department of Biology, New York University, New York, NY 10003, United States

## Abstract

**Summary:**

We introduce an *in silico* PCR (sPCR) method for the assembly of specific genomic regions spanned by PCR primers using raw sequence reads. This allows a user to quickly isolate the exact regions that are abundant in public archives of gene sequences, leveraging the decades of work that have gone into optimizing primer sequences for benchtop PCR. We implement sPCR in sharkmer as a targeted de Bruijn graph assembler seeded with the forward primer sequence and terminated with the reverse primer sequence. This is useful for a variety of routine tasks, including validating the species identity of a dataset, identifying contaminants, and quickly building phylogenies from raw sequence data.

**Availability and implementation:**

sharkmer is written in Rust. Code, instructions for installation and use, tests, and other resources are available in the GitHub repository at https://github.com/caseywdunn/sharkmer and at Zenodo with DOI 10.5281/zenodo.19020708. It can also be installed via bioconda.

## 1 Introduction

With the rapidly growing availability of whole genome sequence data, a common objective is to quickly derive specific sequence regions from raw genomic or transcriptomic reads (Le Bras *et al.* 2016). An investigator may wish, for example, to retrieve a commonly sequenced gene region (i.e. a “barcode”) to compare to existing datasets as a test of species identity. This task, however, can be surprisingly inconsistent and tedious, requiring multiple tools, significant data, and large computational resources. Here we present a new method for the assembly of target sequences from raw reads (*in silico* PCR, sPCR) and implement it in a new tool (sharkmer). This method runs quickly with a single command on a standard laptop, and achieves consistent results even with a relatively small number of reads, by building upon the decades of optimization of PCR primer sequences to amplify specific genome regions.

Previous approaches to deriving specific genome regions from raw reads can be divided into several categories. One approach is to attempt a global assembly of the data (Lin *et al.* 2011), and then search for the desired genome regions in the assembly. This approach is data intensive, and results can be inconsistent given the highly fragmented nature of short read assemblies in combination with variation in genome size and other factors. It is also resource intensive, requiring more memory than is available on most personal computers. Another approach is to map reads to homologous regions of interest and then call a consensus sequence or perform an assembly on the mapped reads (Peterlongo and Chikhi 2012, Allen *et al.* 2015, McCarthy *et al.* 2019). This approach also requires multiple tools, and mapping can have variable success if reference sequences are not available for the sequenced species. Finally, targeted assemblers (Warren and Holt 2011, Allen *et al.* 2018, Crane *et al.* 2022) use an assembly graph seeded with particular sequences, providing a starting point that is extended with the reads. Tools for targeted assembly of organelle genomes, such as NOVOPlasty (Dierckxsens *et al.* 2017) and GetOrganelle (Jin *et al.* 2020), have become very powerful and useful tools for rapid assembly of homologous sequences across specimens, but are restricted to organelles. Some tools combine these different strategies (Kucuk *et al.* 2017, Ahuja *et al.* 2024).

Here we present sharkmer, a kmer counter and targeted assembler that overcomes these challenges by implementing *in silico* PCR (sPCR) on raw reads. A user specifies a pair of primer sequences and raw reads, and sharkmer assembles the sequence spanned by the primers, corresponding to the benchtop PCR product that would be amplified using those same primer sequences. We additionally present a series of preconfigured primer panels for plants, animals, bacteria, and other clades, and use these to validate performance. sPCR as implemented in sharkmer can be run in minutes (or less) on a laptop, and performs well even with relatively few input reads. Specificity to target sequences can be optimized by adjusting parameters with clear parallels to benchtop PCR. sPCR has many potential applications, including (but not limited to) facilitating rapid species and sample identification, reference-free phylogenetic analysis, and identification of contaminants. Previous *in silico* PCR approaches (Yu and Zhang 2011) used primer sequences as queries to pull specific sequences from assemblies, but to our knowledge there is no other approach that uses primer sequences on raw reads directly.

## 2 Design and implementation

**Figure 1:**
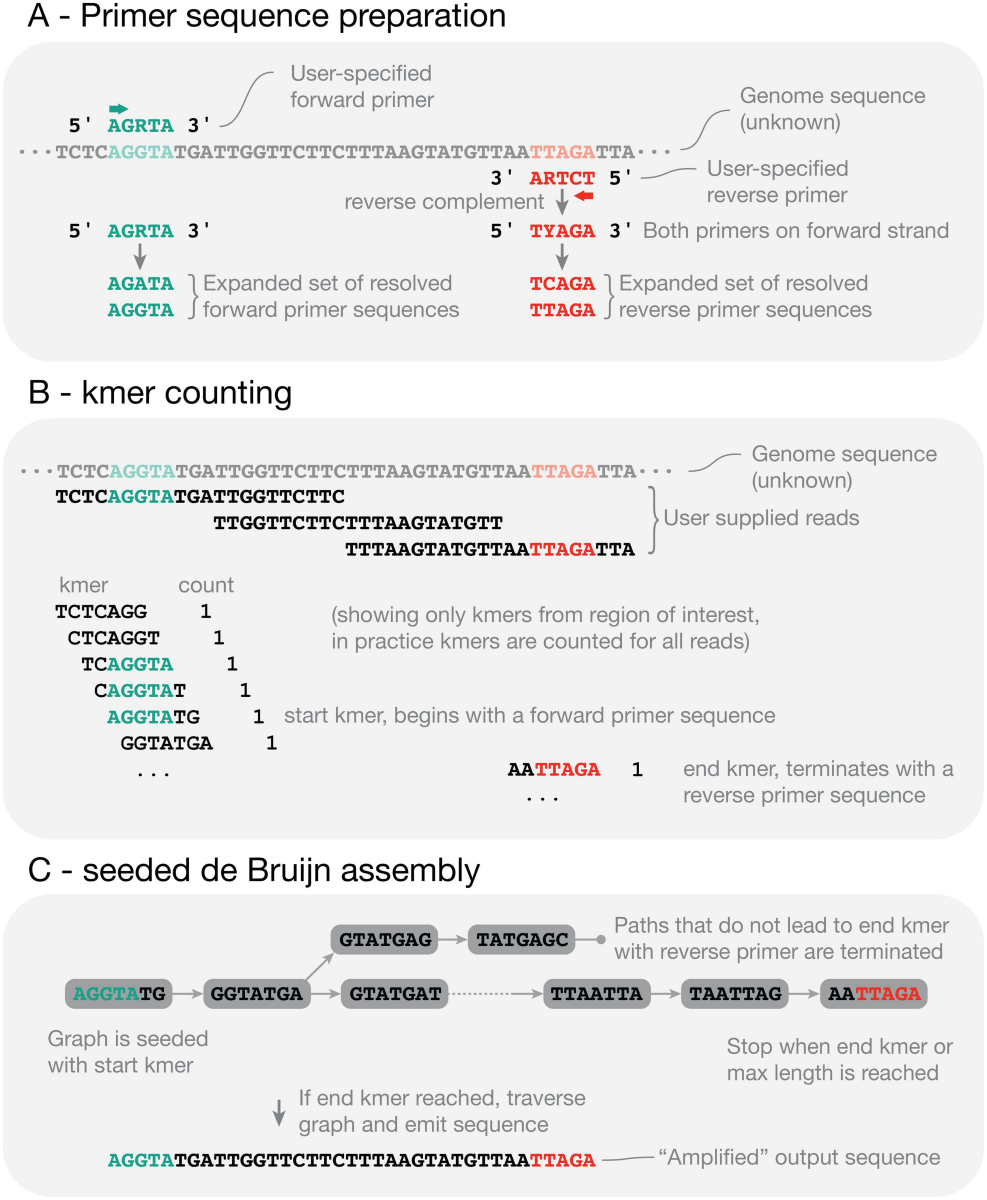
Overview of sPCR in sharkmer, using artificially short sequences to depict workflow. In practice, primer sequences are ∼20 bp, kmers default to 21 bp, Illumina reads are often 150 bp, and amplified regions can be hundreds of bp long. In addition, only a portion of the genome and corresponding kmers are shown, but in practice, there are millions or billions of kmers from across the genome. (A) User defined primers are analyzed to create expanded set of sequences. Not shown here is an initial trimming step that retains only 15 bp from the 3′ end (see text and documentation for details). (B) kmers are counted from reads. (C) de Bruijn graph is assembled using a start kmer as seed. An assembled sequence is emitted only if an end kmer is reached, to add specificity.

sPCR (Fig. 1) is implemented in sharkmer in the Rust programming language. The first step of sPCR is to ingest sequencing reads and count kmers, stored as a hashmap where the key is a 64-bit encoded canonical kmer. The maximum allowable *k* is 31, given that each base is encoded as 2 bits, and that an odd *k* is used to avoid complementary palindromic sequences.

Multiple primer pairs can be specified for each run, allowing the user to quickly isolate multiple genes after a single round of kmer counting. Each primer pair includes a forward and reverse primer, as is standard in benchtop PCR (i.e. primers are on opposite strands with 3′ ends facing one another). We find that sPCR performs well with shorter primers than benchtop PCR. This is because benchtop PCR primers are often lengthened beyond what is necessary for specificity to optimize melting temperature. In sPCR, shorter primers often retain specificity but provide fewer opportunities for mismatch, and more frequently recover the target sequence. By default, sharkmer trims the input primer down to a searched primer of 15 bases, retaining the 3′ end of each primer. Note that the selected *k* for hashing must be as long as, or longer than, the longest searched primers.

The reverse complement of the reverse primer is generated, so that it corresponds to the same strand as the forward primer, and each primer is expanded into a list as follows: (i) ambiguous or degenerate nucleotides are fully resolved in all possible combinations, (ii) new sequences that vary by a specified number of mismatches (2 by default) are added. The new lists of all forward and reverse sequences are then used to generate sets of start and end kmer masks. Start kmers *begin* with a perfect match to any sequence in the forward list, and end kmers *end* with a perfect match to any sequence in the reverse list. If either the start or end list is empty it indicates that the forward and reverse primers were not found, and sPCR is terminated.

For each start kmer, de Bruijn graph assembly is initiated. Unique assembly products that reach an end kmer after min-length nucleotides (default value of 0) and before max-length nucleotides (default value of 10 kbp) are evaluated and returned to the user as a fasta file.

The usage of sPCR, as implemented in sharkmer, has parallels to benchtop PCR in key ways.

First, by using primers as seeds, sPCR directly leverages the decades of work in optimizing PCR primers to work well across species and to span informative gene regions. These primers tend to bind conserved sites that flank variable informative regions and have minimal off-target binding. These properties of primers represent the key advantage of sPCR, as one of the central challenges in deriving sequences from raw reads is optimizing seed sequences used in searching read-space.

Second, because sPCR is primer-based, one can use it to obtain the exact same gene regions that have historically been the target of benchtop PCR amplification. Further, the loci that can most consistently be recovered with sPCR (high copy number mitochondrial and ribosomal genes) reflect some of the most broadly sampled loci in public databases, including cytochrome oxidase 1 (CO1), and ribosomal subunits 16S, 18S, or 28S.

Third, sPCR primer sequences take advantage of degenerate bases to allow for optimization of specificity, as with benchtop PCR primers. The number of allowed mismatches can be adjusted to further control the number of matches, analogous to optimizing melting temperature in benchtop PCR to control non-specific binding. Adjusting the minimum and maximum allowed length for the expected product can also select against off-target products, equivalent to cutting a band of a specific length from a gel after benchtop PCR.

There are several advantages to sPCR over other raw-read workflows. sPCR requires only a single tool (sharkmer), in contrast to complex workflows with multiple dependencies. Additionally, it runs quickly, has minimum computational requirements, and is effective even with relatively low numbers of input reads, as shown in validation experiments.

## 3 Results

Alongside the release of sharkmer, we provide a series of preconfigured panels of primers designed to be useful out-of-the-box for frequently sequenced genes in a broad range of organisms, including Bacteria, angiosperm plants, Metazoa (animals), and within Metazoa, cnidarians, teleost fish, insects, and humans (Supplementary Figures). To validate the performance of sharkmer, we ran these panels against publicly available datasets and found that the program successfully recovers the target product as evaluated using BLAST against the nt database (Supplementary Table 1). When matching gene sequences were present in the database, sharkmer products aligned to the corresponding species with high percent identity (usually >99%). In rare cases (e.g. elongation factor 1-alpha in *Xenia* sp.) sharkmer recovered a product that did not return a strong match to a similar sequence, suggesting that primers amplified an off-target sequence, as can occur in benchtop PCR as well.

To evaluate performance at differing numbers of input reads, we compared the results across a range from 1 thousand to 10 million input reads. With 1 million Illumina 150 bp read as input, we observe that sPCR as implemented in sharkmer is able to complete in a matter of minutes across most datasets (range from <30 s to 7 minutes). Because the computational cost is dominated by the kmer counting step, which is performed once for all primer pairs per search, we observe little additional cost for each added primer pair.

The number of reads needed for success depends on the copy number of the target sequence, the size of the genome, and other factors. For loci that are typically high copy number, such as mitochondrial and nuclear ribosomal, sPCR is able to recover a product with one million reads or fewer (Figures, available as supplementary data at *Bioinformatics* online). In the case of nuclear ribosomal subunit 18S, sPCR is effective with as few as 10 000 reads in some cases. Single copy nuclear genes (e.g. yolk protein 2 *yp2* in *Drosophila melanogaster*) require more reads (10 million) to cover the target sufficiently. Adding reads can increase assembly graph complexity and reduce success, so in some cases there may be an intermediate optimal number of reads (e.g. 16S, CO1 in *Haliclystus octoradiatus*); alternatively, graph complexity may increase before sufficient coverage is obtained so that an sPCR product cannot be found even if there are kmers that contain the primer sequences.

We also validated the ability of sharkmer to correctly assemble targeted mutations in a controlled setting using synthetic reads generated with InSilicoSeq (Gourl *et al.* 2018) for the coral *Porites lutea* (see Supplementary Analyses).

### 3.1 Availability and future directions

Code, instructions for installation and use, tests, and other resources are available in the GitHub repository at https://github.com/caseywdunn/sharkmer and at Zenodo with DOI 10.5281/zenodo.19020708. It can also be installed via bioconda. The project is released under an MIT license. In addition to sPCR, sharkmer also implements incremental kmer counting. This allows users to efficiently count kmers on incrementally larger subsets of data. Applications include assessing the robustness of genome size estimates to sequencing depth.

While not written with metagenomic analyses as the central use-case, sharkmer is able to recover multiple distinct products from samples collected for metagenomics (e.g. 16S products from a coral metagenomics dataset that show local similarity to distinct bacterial taxa with blastn). In addition, we find that by applying primers designed for amplification of potential contaminants (e.g. bacteria, human), sharkmer is able to successfully recover candidate sequences (e.g. bacterial 16S products were recovered from whole genome sequencing of a jellyfish, *Rhopilema esculentum*). These results show sPCR has promising applications in quality control of sequencing reads (verifying species identity, detecting contaminants such as unexpected human or bacterial reads) in addition to serving primary biology goals that require quickly isolating consistent genome regions from raw reads.

## Supplementary Material

btag163_Supplementary_Data

## References

[btag163-B1] Ahuja N , Cao X, Schultz DT et al Giants among cnidaria: large nuclear genomes and rearranged mitochondrial genomes in siphonophores. Genome Biol Evol 2024;16:evae048. 10.1093/gbe/evae048 PMC10980510 38502059

[btag163-B2] Allen JM , Huang DI, Cronk QC et al aTRAM-automated target restricted assembly method: a fast method for assembling loci across divergent taxa from next-generation sequencing data. BMC Bioinformatics 2015;16:98. 10.1186/s12859-015-0515-2 25887972 PMC4380108

[btag163-B3] Allen JM , LaFrance R, Folk RA et al aTRAM 2.0: an improved, flexible locus assembler for NGS data. Evol Bioinform Online 2018;14:1176934318774546. 10.1177/1176934318774546 29881251 PMC5987885

[btag163-B4] Crane CF , Nemacheck JA, Subramanyam S et al SLAG: a program for seeded local assembly of genes in complex genomes. Mol Ecol Resour 2022;22:1999–2017. 10.1111/1755-0998.13580 34995394 PMC9303413

[btag163-B5] Dierckxsens N , Mardulyn P, Smits G. NOVOPlasty: de novo assembly of organelle genomes from whole genome data. Nucleic Acids Res 2017;45:e18. 10.1093/nar/gkw955 28204566 PMC5389512

[btag163-B6] Gourl H , Karlsson-Lindsj O, Hayer J et al Simulating illumina metagenomic data with InSilicoSeq. Bioinformatics 2018;35:521–2. 10.1093/bioinformatics/bty630 PMC6361232 30016412

[btag163-B7] Jin J-J , Yu W-B, Yang J-B et al GetOrganelle: a fast and versatile toolkit for accurate de novo assembly of organelle genomes. Genome Biol 2020;21:241. 10.1186/s13059-020-02154-5 32912315 PMC7488116

[btag163-B8] Kucuk E , Chu J, Vandervalk BP et al Kollector: transcript-informed, targeted de novo assembly of gene loci. Bioinformatics 2017;33:1782–8. 10.1093/bioinformatics/btx078 28186221 PMC5572715

[btag163-B9] Le Bras Y , Collin O, Monjeaud C et al Colib’read on galaxy: a tools suite dedicated to biological information extraction from raw NGS reads. Gigascience 2016;5:9–015. 10.1186/s13742-015-0105-2 26870323 PMC4750246

[btag163-B10] Lin Y , Li J, Shen H et al Comparative studies of de novo assembly tools for next-generation sequencing technologies. Bioinformatics 2011;27:2031–7. 10.1093/bioinformatics/btr319 21636596 PMC3137213

[btag163-B11] McCarthy TW , Chou H-C, Brendel VP. SRAssembler: selective recursive local assembly of homologous genomic regions. BMC Bioinformatics 2019;20:371. 31266441 10.1186/s12859-019-2949-4 PMC6604332

[btag163-B12] Peterlongo P , Chikhi R. Mapsembler, targeted and micro assembly of large NGS datasets on a desktop computer. BMC Bioinformatics 2012;13:48. 10.1186/1471-2105-13-48 22443449 PMC3514201

[btag163-B13] Warren RL , Holt RA. Targeted assembly of short sequence reads. PLoS One 2011;6:e19816. 10.1371/journal.pone.0019816 21589938 PMC3092772

[btag163-B14] Yu B , Zhang C. In silico PCR analysis. *In Silico Tools for Gene Discovery*, 2011, 91–107. 10.1007/978-1-61779-176-5_6 21779992

