# Supplementary material for: Sharkmer: repurposing PCR primers for targeted genome assembly using in silico PCR
Source: Bioinformatics. 2026 Apr 7;42(4):btag163. doi: 10.1093/bioinformatics/btag163 (PMC13090824; doi:10.1093/bioinformatics/btag163)
Supplement: btag163_Supplementary_Data [file btag163_supplementary_data.pdf]

Supplementary Materials

Supplementary Analyses

To validate the ability of `sharkmer` to correctly assemble targeted mutations in a controlled setting, we conducted analyses using synthetic data. These analyses are implemented with Claude Code (Anthropic) in a dedicated repository at https://github.com/caseywdunn/sharkmer_synthetic , which is also archived at Zenodo with DOI 10.5281/zenodo.19021107. We downloaded the *Porites lutea* nuclear genome (GCF_958299795.1, chromosomes only) and mitochondrial genome (OY283138.1) from NCBI. We then created two variant mitochondrial genomes, each with a single nucleotide substitution in the 16S rRNA gene. We used InSilicoSeq with the NovaSeq error model to simulate 150 bp single-end reads: 990,000 reads from the nuclear genome and 10,000 reads from each mitochondrial genome (original and two variants). For each of the three mitochondrial sources, we concatenated its reads with the nuclear reads and ran `sharkmer` in silico PCR with the cnidarian primer panel. This results in datasets with 1% of reads from the mitochondrial genome, in the range of real-world datasets. `sharkmer` successfully assembled 694 bp 16S amplicons from all three read sets. The assembled sequences differed only at the expected substitution sites, confirming that `sharkmer` correctly recovers single nucleotide variants from synthetic read data.

Supplementary Table 1

Table 1: BLAST Results Table

Species	Gene Region	Subject Title	Percent Identity	Alignment Length
Acer_monspessulanum	ITS	Acer monspessulanum external transcribed spacer small subunit ribosomal RNA gene, internal transcribed spacer 1, 5.8S ribosomal RNA gene, internal transcribed spacer 2, and large subunit ribosomal RNA gene, complete sequence	100.000	753
Acer_monspessulanum	atpB-rbcL	Acer opalus chloroplast, complete genome	100.000	812
Acer_monspessulanum	psbA-trnH	Acer opalus chloroplast, complete genome	100.000	508
Acer_monspessulanum	psbM-trnD	Acer opalus chloroplast, complete genome	100.000	699
Acer_monspessulanum	trnC-ycf6	Acer opalus chloroplast, complete genome	100.000	593
Acer_monspessulanum	trnL-F	Acer monspessulanum chloroplast, complete genome	100.000	418
Acer_monspessulanum	ycf6-psbM	Acer opalus chloroplast, complete genome	100.000	937
Agalma_elegans	16S	Agalma elegans 16S ribosomal RNA gene, partial sequence; mitochondrial	100.000	527
Agalma_elegans	18S	Uncultured eukaryote clone SGYX1382 18S ribosomal RNA gene, partial sequence	99.664	1784
Agalma_elegans	28S	Halistemma rubrum voucher Yale Peabody Museum No. 35359 28S large subunit ribosomal RNA gene, partial sequence	99.064	3205
Agalma_elegans	28S-v2	Halistemma rubrum voucher Yale Peabody Museum No. 35359 28S large subunit ribosomal RNA gene, partial sequence	97.602	417
Agalma_elegans	CO1	Agalma elegans voucher Hy063.1.3 cytochrome oxidase subunit I (COI) gene, partial cds; mitochondrial	98.963	675
Agalma_elegans	ITS	Uncultured eukaryote clone S32T_08 small subunit ribosomal RNA gene, partial sequence; internal transcribed spacer 1, 5.8S ribosomal RNA gene, and internal transcribed spacer 2, complete sequence; and large subunit ribosomal RNA gene, partial sequence	99.690	645
Agalma_elegans	ITS-v2	Uncultured eukaryote clone S32T_08 small subunit ribosomal RNA gene, partial sequence; internal transcribed spacer 1, 5.8S ribosomal RNA gene, and internal transcribed spacer 2, complete sequence; and large subunit ribosomal RNA gene, partial sequence	99.677	619

Table 1: BLAST Results Table *(continued)*

**Species**	**Gene Region**	**Subject Title**	**Percent Identity**	**Alignment Length**
Bargmannia_gutmetagenome	18S-V3	Pezizomycotina sp. FU16 gene for 18S ribosomal RNA, partial sequence	99.359	156
Bargmannia_gutmetagenome	18S-V7	Thysanoessa raschii voucher E54 small subunit ribosomal RNA gene, partial sequence	100.000	194
Coral_metagenome	16S-341F-783Rabc	Apicomplexa sp. WK-2018_Corallicola apicoplast, complete genome	100.000	433
Coral_metagenome	16S-341F-785R	Apicomplexa sp. WK-2018_Corallicola apicoplast, complete genome	100.000	294
Coral_metagenome	16S-515F-806R	Uncultured prokaryote clone New.CleanUp.ReferenceOTU242471 16S ribosomal RNA gene, partial sequence	100.000	283
Coral_metagenome	16S-515F-806RB	Uncultured prokaryote clone New.CleanUp.ReferenceOTU242471 16S ribosomal RNA gene, partial sequence	100.000	283
Coral_metagenome	16S-PRK341F-PRK806R	Apicomplexa sp. WK-2018_Corallicola apicoplast, complete genome	100.000	294
Covercrop_rhizosphere	16S-515F-806R	Sphingobacteriales bacterium strain Swamp196 16S ribosomal RNA gene, partial sequence	100.000	280
Covercrop_rhizosphere	16S-515F-806RB	Sphingobacteriales bacterium strain Swamp196 16S ribosomal RNA gene, partial sequence	100.000	280
Covercrop_rhizosphere	16S-68F-783Rabc	Uncultured bacterium clone 2007WL_16S_OTU_1946 16S ribosomal RNA gene, partial sequence	97.525	202
Drosophila_melanogaster	12S	Drosophila melanogaster isolate dmeA_15_F0 chromosome 3L	99.275	138
Drosophila_melanogaster	18S	PREDICTED: Drosophila erecta small subunit ribosomal RNA (LOC113564718), rRNA	100.000	661
Drosophila_melanogaster	18S-v2	Drosophila melanogaster 18S ribosomal RNA (18SrRNA:CR45838), rRNA	100.000	1977
Drosophila_melanogaster	CO1	Drosophila melanogaster isolate VAITC7139a cytochrome c oxidase subunit I (COX1) gene, partial cds; mitochondrial	100.000	352

Table 1: BLAST Results Table *(continued)*

**Species**	**Gene Region**	**Subject Title**	**Percent Identity**	**Alignment Length**
Drosophila_melanogaster	CO1-v2	Drosophila melanogaster isolate Canton-S CS-4 cytochrome c oxidase subunit I (COX1) gene, partial cds; tRNA-Leu gene, complete sequence; cytochrome c oxidase subunit II (COX2) gene, complete cds; tRNA-Lys and tRNA-Asp genes, complete sequence; ATPase 8 (ATP8) and ATPase 6 (ATP6) genes, complete cds; and cytochrome c oxidase subunit III (COX3) gene, partial cds; mitochondrial	100.000	859
Drosophila_melanogaster	CO2	Drosophila melanogaster isolate Canton-S CS-4 cytochrome c oxidase subunit I (COX1) gene, partial cds; tRNA-Leu gene, complete sequence; cytochrome c oxidase subunit II (COX2) gene, complete cds; tRNA-Lys and tRNA-Asp genes, complete sequence; ATPase 8 (ATP8) and ATPase 6 (ATP6) genes, complete cds; and cytochrome c oxidase subunit III (COX3) gene, partial cds; mitochondrial	100.000	291
Drosophila_melanogaster	CO2-v2	Drosophila melanogaster isolate Canton-S CS-4 cytochrome c oxidase subunit I (COX1) gene, partial cds; tRNA-Leu gene, complete sequence; cytochrome c oxidase subunit II (COX2) gene, complete cds; tRNA-Lys and tRNA-Asp genes, complete sequence; ATPase 8 (ATP8) and ATPase 6 (ATP6) genes, complete cds; and cytochrome c oxidase subunit III (COX3) gene, partial cds; mitochondrial	100.000	777
Drosophila_melanogaster	CytB	Drosophila melanogaster cell line Del mitochondrion, partial genome	100.000	403
Drosophila_melanogaster	ND1	Drosophila melanogaster cell line Del mitochondrion, partial genome	100.000	240
Drosophila_melanogaster	ND5	Drosophila melanogaster cell line Del mitochondrion, partial genome	100.000	301
Drosophila_melanogaster	Yp2	Drosophila melanogaster strain rover (forR) chromosome X	99.060	745
Drosophila_sechellia	12S	Drosophila sechellia voucher DSECH20161109 mitochondrion, complete genome	100.000	419

Table 1: BLAST Results Table *(continued)*

Species	Gene Region	Subject Title	Percent Identity	Alignment Length
Drosophila__sechellia	16S	Drosophila sechellia voucher DSECH20161109 mitochondrion, complete genome	100.000	380
Drosophila__sechellia	16S-v2	Drosophila sechellia voucher DSECH20161109 mitochondrion, complete genome	100.000	541
Drosophila__sechellia	18S	PREDICTED: Drosophila mauritiana small subunit ribosomal RNA (LOC117148991), rRNA	100.000	661
Drosophila__sechellia	18S-v2	PREDICTED: Drosophila mauritiana small subunit ribosomal RNA (LOC117148991), rRNA	100.000	1977
Drosophila__sechellia	CO1	Drosophila sechellia voucher DSECH20161109 mitochondrion, complete genome	100.000	352
Drosophila__sechellia	CO1-v2	Drosophila sechellia voucher DSECH20161109 mitochondrion, complete genome	100.000	859
Drosophila__sechellia	CO2-v2	Drosophila sechellia voucher DSECH20161109 mitochondrion, complete genome	100.000	777
Drosophila__sechellia	CytB	Drosophila sechellia voucher DSECH20161109 mitochondrion, complete genome	100.000	403
Drosophila__sechellia	ITS	Drosophila melanogaster chromosome X; Y rDNA sequence	100.000	75
Drosophila__sechellia	NADH	Drosophila sechellia mitochondrion, complete genome	100.000	550
Drosophila__sechellia	ND1	Drosophila sechellia voucher DSECH20161109 mitochondrion, complete genome	100.000	240
Drosophila__sechellia	ND4	Drosophila sechellia voucher DSECH20161109 mitochondrion, complete genome	100.000	241
Drosophila__sechellia	ND5	PREDICTED: Drosophila sechellia small subunit ribosomal RNA (LOC116803316), rRNA	100.000	321
Drosophila__tanythrix	12S	Drosophila adunca voucher 105818 16S ribosomal RNA gene, partial sequence; tRNA-Val gene, complete sequence; and 12S ribosomal RNA gene, partial sequence; mitochondrial	99.511	409
Drosophila__tanythrix	16S	Drosophila paracracens voucher 202556 16S ribosomal RNA gene, partial sequence; mitochondrial	100.000	379

Table 1: BLAST Results Table *(continued)*

**Species**	**Gene Region**	**Subject Title**	**Percent Identity**	**Alignment Length**
Drosophila_tanythrix	16S-v2	Drosophila orthoptera voucher 202540 16S ribosomal RNA gene, partial sequence; mitochondrial	100.000	510
Drosophila_tanythrix	18S	Hirtodrosophila cameraria genome assembly, chromosome: 2	99.546	661
Drosophila_tanythrix	18S-v2	PREDICTED: Drosophila grimshawi small subunit ribosomal RNA (LOC122322811), rRNA	99.798	1976
Drosophila_tanythrix	CO1	Drosophila tanythrix isolate 572 cytochrome c oxidase subunit I (COX1) gene, partial cds; mitochondrial	99.432	352
Drosophila_tanythrix	CO1-v2	Drosophila diamphidiopoda voucher 200785 cytochrome oxidase subunit 1 (CO1) gene, partial cds; mitochondrial	97.346	829
Drosophila_tanythrix	CO2	Pseudomonas fragi strain NMC25, complete genome	100.000	331
Drosophila_tanythrix	CO2-v2	Drosophila yooni voucher 202550 cytochrome oxidase subunit II (CO2) gene, partial cds; mitochondrial	99.158	713
Drosophila_tanythrix	CytB	Drosophila adunca voucher 105818 cytochrome b (cytb) gene, partial cds; mitochondrial	95.250	400
Drosophila_tanythrix	ITS	Hirtodrosophila cameraria genome assembly, chromosome: 2	100.000	76
Drosophila_tanythrix	NADH	Drosophila tanythrix voucher 202520 NADH dehydrogenase subunit 2 (ND2) gene, partial cds; mitochondrial	99.423	520
Drosophila_tanythrix	ND1	Phortica variegata voucher PVARI20161110M mitochondrion, complete genome	92.500	240
Drosophila_tanythrix	ND4	Sarcophila rasnitzyni mitochondrion, complete genome	85.185	243
Drosophila_tanythrix	ND5	PREDICTED: Drosophila grimshawi NADH-ubiquinone oxidoreductase chain 5-like (LOC116806149), mRNA	95.681	301
Engraulis_encrasicolus	12S	Engraulis encrasicolus isolate F74 small subunit ribosomal RNA gene, partial sequence; mitochondrial	100.000	417
Engraulis_encrasicolus	16S	Engraulis encrasicolus isolate DM247 mitochondrion, complete genome	100.000	605

Table 1: BLAST Results Table *(continued)*

Species	Gene Region	Subject Title	Percent Identity	Alignment Length
Engraulis__encrasicolus	18S	Anchoa hepsetus voucher USNM:FISH:419965 small subunit ribosomal RNA gene, partial sequence; internal transcribed spacer 1, 5.8S ribosomal RNA gene, and internal transcribed spacer 2, complete sequence; and large subunit ribosomal RNA gene, partial sequence	97.352	491
Engraulis__encrasicolus	CO1	Engraulis encrasicolus isolate DM247 mitochondrion, complete genome	100.000	685
Euphorbia__nicaeensis	ITS	Euphorbia nicaeensis isolate 13368 internal transcribed spacer 1, partial sequence; 5.8S ribosomal RNA gene and internal transcribed spacer 2, complete sequence; and large subunit ribosomal RNA gene, partial sequence	99.004	703
Euphorbia__nicaeensis	atpB-rbcL	Euphorbia esula cultivar 1984-ND001 chloroplast, complete genome	96.252	827
Euphorbia__nicaeensis	psbA-trnH	Euphorbia tirucalli voucher N.Wei 1064 (HIB) chloroplast, complete genome	93.902	82
Euphorbia__nicaeensis	trnC-ycf6	Euphorbia ebracteolata chloroplast, complete genome	93.229	384
Euphorbia__nicaeensis	trnL-F	Euphorbia nicaeensis isolate 14676 trnT-trnF intergenic spacer, partial sequence; chloroplast	99.767	429
Euphorbia__nicaeensis	ycf6-psbM	Euphorbia altotibetica chloroplast, complete genome	96.624	237
Gryllus__bimaculatus	12S	Gryllus bimaculatus mitochondrion, complete genome	99.291	423
Gryllus__bimaculatus	16S	Gryllus bimaculatus clone G2225 16S ribosomal RNA gene, partial sequence; mitochondrial	100.000	376
Gryllus__bimaculatus	16S-v2	Gryllus bimaculatus mRNA, GBcontig00008	100.000	36
Gryllus__bimaculatus	18S	Gryllus bimaculatus mRNA, GBcontig31064	100.000	709
Gryllus__bimaculatus	18S-v2	Gryllus bimaculatus mRNA, GBcontig31064	100.000	1325
Gryllus__bimaculatus	28S	Acheta domesticus large subunit ribosomal RNA gene, partial sequence	96.721	305
Gryllus__bimaculatus	CO1	Gryllus bimaculatus isolate H088 cytochrome oxidase subunit I gene, partial cds; mitochondrial	99.671	304
Gryllus__bimaculatus	CO1-v2	Gryllus bimaculatus mitochondrion, complete genome	99.532	855

Table 1: BLAST Results Table *(continued)*

**Species**	**Gene Region**	**Subject Title**	**Percent Identity**	**Alignment Length**
Gryllus_bimaculatus	CO2	Gryllus bimaculatus mitochondrion, complete genome	99.653	288
Gryllus_bimaculatus	CytB	Wolbachia endosymbiont (group A) of Epagoge grotiana isolate 26617 genome assembly, chromosome: 1	99.396	497
Gryllus_bimaculatus	ITS	Gryllus bimaculatus voucher WG23 28S ribosomal RNA gene, partial sequence	100.000	1266
Gryllus_bimaculatus	NADH	Gryllus bimaculatus mitochondrion, complete genome	99.081	544
Gryllus_bimaculatus	ND1	Gryllus bimaculatus mitochondrion, complete genome	99.583	240
Gryllus_bimaculatus	ND4	Gryllus bimaculatus mitochondrion, complete genome	97.925	241
Gryllus_bimaculatus	ND5	Gryllus bimaculatus mitochondrion, complete genome	99.320	294
Haliclystus_octoradiatus	16S	Haliclystus octoradiatus genome assembly, organelle: mitochondrion	100.000	578
Haliclystus_octoradiatus	18S	Haliclystus stejnegeri isolate 06JAP42-2 small subunit ribosomal RNA gene, partial sequence	100.000	1664
Haliclystus_octoradiatus	28S	Haliclystus octoradiatus genome assembly, chromosome: 6	100.000	3242
Haliclystus_octoradiatus	28S-v2	Haliclystus octoradiatus isolate DF12 28S large subunit ribosomal RNA gene, partial sequence	100.000	420
Haliclystus_octoradiatus	CO1	Haliclystus octoradiatus genome assembly, organelle: mitochondrion	99.855	688
Haliclystus_octoradiatus	ITS	Haliclystus octoradiatus isolate DF12 18S small subunit ribosomal RNA gene, partial sequence; internal transcribed spacer 1, 5.8S ribosomal RNA gene, and internal transcribed spacer 2, complete sequence; and 28S large subunit ribosomal RNA gene, partial sequence	99.694	653
Haliclystus_octoradiatus	ITS-v2	Haliclystus octoradiatus isolate DF12 18S small subunit ribosomal RNA gene, partial sequence; internal transcribed spacer 1, 5.8S ribosomal RNA gene, and internal transcribed spacer 2, complete sequence; and 28S large subunit ribosomal RNA gene, partial sequence	99.694	653

Table 1: BLAST Results Table *(continued)*

**Species**	**Gene Region**	**Subject Title**	**Percent Identity**	**Alignment Length**
Heliconius__pachinus	12S	Heliconius pachinus mitochondrion, complete genome	100.000	417
Heliconius__pachinus	16S	Heliconius pachinus voucher 3035; mitochondrial	100.000	380
Heliconius__pachinus	18S-v2	Heliconius sara genome assembly, chromosome: 6	100.000	1885
Heliconius__pachinus	CO1	Heliconius pachinus voucher p520-STRI cytochrome oxidase subunit I (COI) gene, partial cds; mitochondrial	100.000	304
Heliconius__pachinus	CO1-v2	Heliconius pachinus voucher TX520 cytochrome oxidase subunit I (COI) gene, partial cds; tRNA-Leu (trnL) gene, complete sequence; and cytochrome oxidase subunit II (COII) gene, complete cds; mitochondrial	100.000	840
Heliconius__pachinus	CO2	Heliconius pachinus voucher TX595 cytochrome oxidase subunit I (COI) gene, partial cds; tRNA-Leu (trnL) gene, complete sequence; and cytochrome oxidase subunit II (COII) gene, complete cds; mitochondrial	100.000	288
Heliconius__pachinus	CytB	Heliconius pachinus mitochondrion, complete genome	99.752	403
Heliconius__pachinus	ND1	Heliconius pachinus mitochondrion, complete genome	100.000	240
Heliconius__pachinus	ND4	Heliconius pachinus mitochondrion, complete genome	99.585	241
Heliconius__pachinus	ND5	Heliconius cydno mitochondrion, complete genome	99.683	315
Hirundichthys__speculiger	12S	Diaphus theta mitochondrion, complete genome	96.471	425
Hirundichthys__speculiger	16S	Diaphus mollis mitochondrial gene for 16S rRNA	96.283	565
Hirundichthys__speculiger	18S	Diaphus fulgens voucher SIO:Marine Vertebrates:10__165 small subunit ribosomal RNA gene, partial sequence; internal transcribed spacer 1, 5.8S ribosomal RNA gene, and internal transcribed spacer 2, complete sequence; and large subunit ribosomal RNA gene, partial sequence	100.000	477
Homo__sapiens	mt10360-12226	Homo sapiens isolate 1039 mitochondrion, complete genome	100.000	1853

Table 1: BLAST Results Table *(continued)*

**Species**	**Gene Region**	**Subject Title**	**Percent Identity**	**Alignment Length**
Homo_sapiens	mt14898-151	Homo sapiens isolate HGDP00950 polymorphic numt genomic sequence; nuclear copy of mitochondrial sequence	99.447	1809
Homo_sapiens	mt16488-1677	Homo sapiens isolate Nvh1631 haplogroup Y1a-16189! mitochondrion, complete genome	100.000	1670
Homo_sapiens	mt6511-9220	Homo sapiens isolate PNG51 haplogroup M28a+204 mitochondrion, complete genome	100.000	2694
Homo_sapiens	mt8910-10648	Homo sapiens isolate UP2013 haplogroup L3b1a11 mitochondrion, complete genome	100.000	1725
Liriodendron_tulipifera	atpB-rbcL	Liriodendron tulipifera chloroplast, complete genome	100.000	812
Liriodendron_tulipifera	psbA-trnH	Liriodendron tulipifera chloroplast, complete genome	100.000	520
Liriodendron_tulipifera	psbM-trnD	Liriodendron tulipifera chloroplast, complete genome	100.000	1287
Liriodendron_tulipifera	rpl36-infA-rps8	Liriodendron tulipifera chloroplast, complete genome	100.000	552
Liriodendron_tulipifera	trnC-ycf6	Liriodendron tulipifera chloroplast, complete genome	100.000	1099
Liriodendron_tulipifera	trnK-rps16	Liriodendron tulipifera chloroplast, complete genome	100.000	657
Liriodendron_tulipifera	trnL-F	Liriodendron tulipifera chloroplast, complete genome	100.000	434
Liriodendron_tulipifera	ycf6-psbM	Liriodendron tulipifera chloroplast, complete genome	100.000	927
Morbakka_sp	16S	Morbakka virulenta voucher L5 large subunit ribosomal RNA gene, partial sequence; mitochondrial	99.550	444
Morbakka_sp	16S-27F-338R	Photobacterium jeanii strain R-21419 16S ribosomal RNA gene, partial sequence	100.000	343
Morbakka_sp	16S-341F-783Rabc	Bacterium strain 2-5 16S ribosomal RNA gene, partial sequence	100.000	455
Morbakka_sp	16S-341F-785R	Bacterium strain 2-5 16S ribosomal RNA gene, partial sequence	100.000	457
Morbakka_sp	16S-515F-806R	Bacterium strain 2-5 16S ribosomal RNA gene, partial sequence	100.000	283
Morbakka_sp	16S-515F-806RB	Bacterium strain 2-5 16S ribosomal RNA gene, partial sequence	100.000	283

Table 1: BLAST Results Table *(continued)*

**Species**	**Gene Region**	**Subject Title**	**Percent Identity**	**Alignment Length**
Morbakka_sp	16S-515F-Y-926R	Bacterium strain 2-5 16S ribosomal RNA gene, partial sequence	100.000	403
Morbakka_sp	16S-68F-518R	Photobacterium jeanii strain R-21419 16S ribosomal RNA gene, partial sequence	100.000	489
Morbakka_sp	16S-799F-1193R	Bacterium strain 2-5 16S ribosomal RNA gene, partial sequence	100.000	407
Morbakka_sp	16S-967F-1391R	Bacterium strain 2-5 16S ribosomal RNA gene, partial sequence	100.000	435
Morbakka_sp	16S-B969F-BA1406R	Bacterium strain 2-5 16S ribosomal RNA gene, partial sequence	100.000	434
Morbakka_sp	16S-PRK341F-PRK806R	Bacterium strain 2-5 16S ribosomal RNA gene, partial sequence	100.000	459
Morbakka_sp	16S-V2f-V3r	Bacterium strain 2-5 16S ribosomal RNA gene, partial sequence	100.000	424
Morbakka_sp	18S	Morbakka virulenta isolate BB035 18S small subunit ribosomal RNA gene, partial sequence	99.942	1734
Morbakka_sp	28S	Morbakka virulenta isolate BB035 28S large subunit ribosomal RNA gene, partial sequence	99.930	2838
Morbakka_sp	28S-v2	Morbakka virulenta isolate BB035 28S large subunit ribosomal RNA gene, partial sequence	99.543	438
Morbakka_sp	CO1	Morbakka virulenta Hir111210 mitochondrial COI gene for cytochrome c oxidase subunit 1, partial cds	94.505	637
Nomeus_gronovii	12S	Nomeus gronovii KBF-I 1479 mitochondrial genes for 12S rRNA, tRNA-Val, 16S rRNA, partial and complete sequence	99.761	419
Nomeus_gronovii	16S	Cubiceps squamiceps mitochondrion, complete genome	97.195	606
Nomeus_gronovii	18S	Cubiceps paradoxus voucher SIO:Marine Vertebrates:19_76 small subunit ribosomal RNA gene, partial sequence; internal transcribed spacer 1, 5.8S ribosomal RNA gene, and internal transcribed spacer 2, complete sequence; and large subunit ribosomal RNA gene, partial sequence	99.788	472
Nomeus_gronovii	CO1	Nomeus gronovii voucher Smith 255.7 #3 cytochrome oxidase subunit 1 (COI) gene, partial cds; mitochondrial	99.540	652

Table 1: BLAST Results Table *(continued)*

Species	Gene Region	Subject Title	Percent Identity	Alignment Length
Opuntia__comonduensis	ITS	Opuntia bonaerensis voucher F.Font 423 (BAF) external transcribed spacer small subunit ribosomal RNA gene, internal transcribed spacer 1, 5.8S ribosomal RNA gene, internal transcribed spacer 2, and large subunit ribosomal RNA gene, complete sequence	99.418	687
Opuntia__comonduensis	rpl36-infA-rps8	Opuntia sulphurea chloroplast, complete genome	98.913	92
Opuntia__comonduensis	trnC-ycf6	Opuntia quimilo chloroplast, complete genome	99.068	644
Opuntia__comonduensis	trnL-F	Opuntia tehuacana isolate CMOT30 trnL-trnF intergenic spacer, partial sequence; chloroplast	100.000	455
Porites__lutea	18S	Porites profundus partial 18S rRNA gene, specimen voucher MY293	100.000	1782
Porites__lutea	28S	Blastomussa wellsi genome assembly, chromosome: 7	98.684	76
Porites__lutea	28S-v2	Porites compressa isolate PC5b 28S large subunit ribosomal RNA gene, partial sequence	99.767	430
Porites__lutea	ITS	Goniopora gracilis isolate 3 18S ribosomal RNA gene, partial sequence; internal transcribed spacer 1, 5.8S ribosomal RNA gene, and internal transcribed spacer 2, complete sequence; and 28S ribosomal RNA gene, partial sequence	99.292	847
Porites__lutea	ITS-v2	Goniopora gracilis isolate 3 18S ribosomal RNA gene, partial sequence; internal transcribed spacer 1, 5.8S ribosomal RNA gene, and internal transcribed spacer 2, complete sequence; and 28S ribosomal RNA gene, partial sequence	99.286	700
Rhopilema__esculentum	16S	Rhopilema esculentum isolate Bohai02 16S ribosomal RNA gene, partial sequence; mitochondrial	100.000	505
Rhopilema__esculentum	16S-27F-338R	Uncultured marine bacterium clone BS__OffshoreExp__T48h__A__G7 16S ribosomal RNA gene, partial sequence	97.953	342
Rhopilema__esculentum	16S-341F-783Rabc	Uncultured marine bacterium clone BS__OffshoreExp__T48h__A__G7 16S ribosomal RNA gene, partial sequence	97.588	456

Table 1: BLAST Results Table *(continued)*

**Species**	**Gene Region**	**Subject Title**	**Percent Identity**	**Alignment Length**
Rhopilema_esculentum	16S-341F-785R	Uncultured marine bacterium clone BS_OffshoreExp_T48h_A_G7 16S ribosomal RNA gene, partial sequence	97.598	458
Rhopilema_esculentum	16S-515F-806R	Uncultured marine bacterium clone B1G5 16S ribosomal RNA gene, partial sequence	98.582	282
Rhopilema_esculentum	16S-515F-806RB	Uncultured marine bacterium clone B1G5 16S ribosomal RNA gene, partial sequence	98.582	282
Rhopilema_esculentum	16S-515F-Y-926R	Uncultured marine bacterium clone B1G5 16S ribosomal RNA gene, partial sequence	98.281	349
Rhopilema_esculentum	16S-68F-518R	Uncultured marine bacterium clone BS_CoastalExp_T55h_A_H5 16S ribosomal RNA gene, partial sequence	97.930	483
Rhopilema_esculentum	16S-967F-1391R	Uncultured marine bacterium clone BS_CoastalExp_T55h_A_B3 16S ribosomal RNA gene, partial sequence	99.534	429
Rhopilema_esculentum	16S-B969F-BA1406R	Uncultured marine bacterium clone BS_CoastalExp_T55h_A_B3 16S ribosomal RNA gene, partial sequence	99.533	428
Rhopilema_esculentum	16S-PRK341F-PRK806R	Uncultured marine bacterium clone BS_OffshoreExp_T48h_A_G7 16S ribosomal RNA gene, partial sequence	97.609	460
Rhopilema_esculentum	16S-V2f-V3r	Rhopilema esculentum mitochondrion, complete genome	99.510	204
Rhopilema_esculentum	18S	Rhopilema esculentum isolate M0D05980Z 18S small subunit ribosomal RNA gene, partial sequence	100.000	1766
Rhopilema_esculentum	28S	Chrysaora pacifica voucher AZ08-2 18S ribosomal RNA gene, internal transcribed spacer 1, 5.8S ribosomal RNA gene, internal transcribed spacer 2, 28S ribosomal RNA gene, and intergenic spacer, complete sequence	95.343	2577
Rhopilema_esculentum	28S-v2	Rhopilema esculentum isolate M0D05980Z 28S large subunit ribosomal RNA gene, partial sequence	100.000	426
Rhopilema_esculentum	CO1	Rhopilema esculentum mitochondrial partial COI gene for cytochrome oxidase subunit 1, specimen voucher RES2	100.000	658
Rhopilema_esculentum	EF1A	Sparus aurata genome assembly, chromosome: 7	74.653	288

Table 1: BLAST Results Table *(continued)*

Species	Gene Region	Subject Title	Percent Identity	Alignment Length
Rhopilema_esculentum	ITS-v2	Rhopilema esculentum genes for 18S rRNA, internal transcribed spacer 1 and 5.8S rRNA, partial and complete sequence, isolate: KUR66	100.000	402
Seawater_metagenome	16S-515F-806R	Colwellia sp. strain S3V2 16S ribosomal RNA gene, partial sequence	100.000	283
Seawater_metagenome	16S-515F-806RB	Colwellia sp. strain S3V2 16S ribosomal RNA gene, partial sequence	100.000	283
Seawater_metagenome	16S-515F-Y-926R	Bacterium AK42 gene for 16S ribosomal RNA, partial sequence	99.504	403
Seawater_metagenome	16S-68F-783Rabc	Uncultured bacterium clone SeaWat_51049 16S ribosomal RNA gene, partial sequence	99.010	202
Sphaeronectes_gutmetagenome	18S-V3	Uroglenopsis turfosa isolate UK-81 18S ribosomal RNA gene, partial sequence	100.000	154
Sphaeronectes_gutmetagenome	18S-V5-V7L	Sphaeronectes christiansonae isolate 2 18S ribosomal RNA gene, complete sequence	98.851	261
Sphaeronectes_gutmetagenome	18S-V5-V7S	Sphaeronectes haddocki 18S ribosomal RNA gene, complete sequence	100.000	142
Sphaeronectes_gutmetagenome	18S-V7	Chelophyes sp. 242_UnivF-1183 clone 242_UnivF-1183 18S ribosomal RNA gene, partial sequence	100.000	172
Sphaeronectes_gutmetagenome	18S-V7p-V8	Hippopodiiae sp. 1_UnivF-1183 clone 1_UnivF-1183 18S ribosomal RNA gene, partial sequence	100.000	203
Sphaeronectes_gutmetagenome	18S-V9	Sphaeronectes haddocki 18S ribosomal RNA gene, complete sequence	100.000	129
Xenia_sp	16S	PREDICTED: Xenia sp. Carnegie-2017 NADH-ubiquinone oxidoreductase chain 2-like (LOC124455316), mRNA	100.000	621
Xenia_sp	18S	PREDICTED: Xenia sp. Carnegie-2017 small subunit ribosomal RNA (LOC124456144), rRNA	99.889	1807
Xenia_sp	28S	PREDICTED: Xenia sp. Carnegie-2017 large subunit ribosomal RNA (LOC124456156), rRNA	99.629	3237
Xenia_sp	28S-v2	Ovabunda biseriata voucher ZMTAU:Co34172 large subunit ribosomal RNA gene, partial sequence	100.000	414

Table 1: BLAST Results Table *(continued)*

Species	Gene Region	Subject Title	Percent Identity	Alignment Length
Xenia_sp	CO1	Ovabunda faraunenesis voucher ZMTAU CO34051 cytochrome oxidase subunit II (cox2) and cytochrome oxidase subunit I (cox1) genes, partial cds; mitochondrial	100.000	688
Xenia_sp	EF1A	PREDICTED: Xenia sp. Carnegie-2017 probable ATP-dependent DNA helicase HFM1 (LOC124456206), mRNA	100.000	73
Xenia_sp	ITS	Ovabunda biseriata voucher ZMTAU:36782 18S ribosomal RNA gene, partial sequence; internal transcribed spacer 1, 5.8S ribosomal RNA gene, and internal transcribed spacer 2, complete sequence; and 28S ribosomal RNA gene, partial sequence	100.000	811
Xenia_sp	ITS-v2	Ovabunda biseriata voucher ZMTAU:36782 18S ribosomal RNA gene, partial sequence; internal transcribed spacer 1, 5.8S ribosomal RNA gene, and internal transcribed spacer 2, complete sequence; and 28S ribosomal RNA gene, partial sequence	100.000	748

Supplementary Figures

sPCR on preconfigured primer panels for a wide range of organisms. SRA numbers provided at: https://github.com/caseywdunn/sharkmer/tests/config.yaml

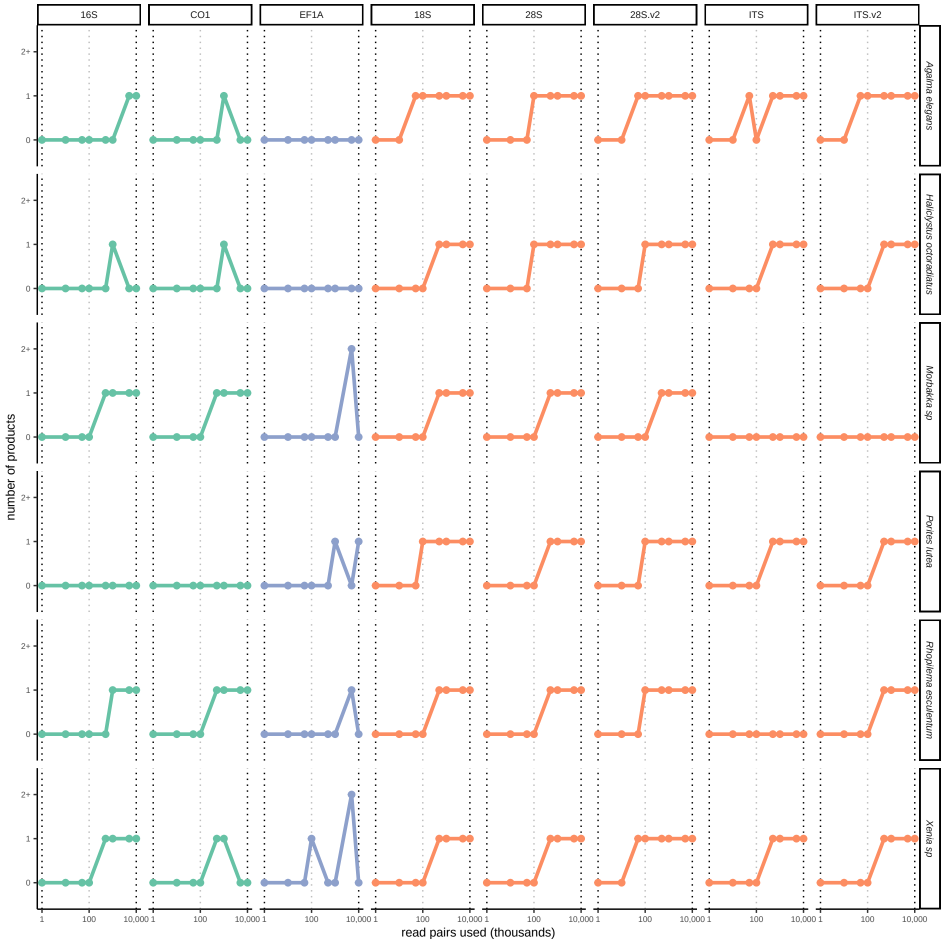

Figure S1: sPCR on a preconfigured primer panel for commonly sequenced genes in Cnidaria. sPCR, as implemented in `sharkmer`, was performed on six publicly available cnidarian datasets (SRA accession numbers: *Agalma elegans*, SRR25099394; *Haliclystus octoradiatus*, ERR6745737; *Morbakka sp.*, SRR25627438; *Porites lutea*, ERR571460; *Rhopilema esculentum*, SRR8617500; *Xenia sp.*, SRR9278435). The preconfigured primer panel includes two mitochondrial loci (16S and CO1, green), one nuclear locus (EF1A, purple), and three nuclear ribosomal loci (18S, 28S, and ITS, orange), with an alternate primer pair provided for each of 28S and ITS. sPCR was run with an increasing number of read pairs, as shown on the x-axis (150 bp read pairs shown in thousands), and the number of products (0, 1, or 2+) is reported on the y-axis.

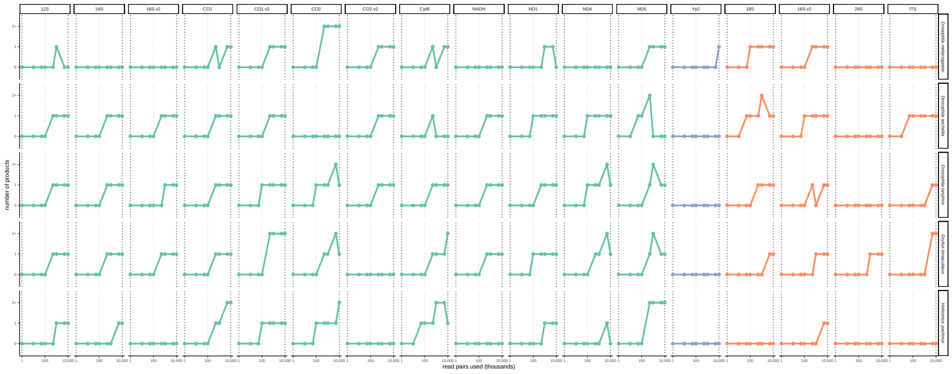

Figure S2: sPCR on the preconfigured primer panel for insects. teal = mitochondrial loci, purple = nuclear, orange = nuclear ribosomal.

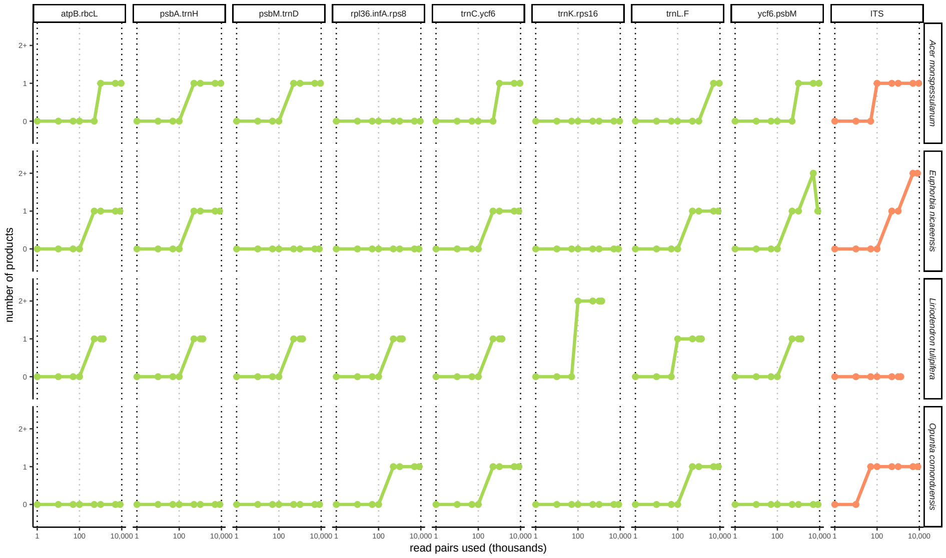

Figure S3: sPCR on the preconfigured primer panel for angiosperm plants. green = chloroplast loci, orange = nuclear ribosomal

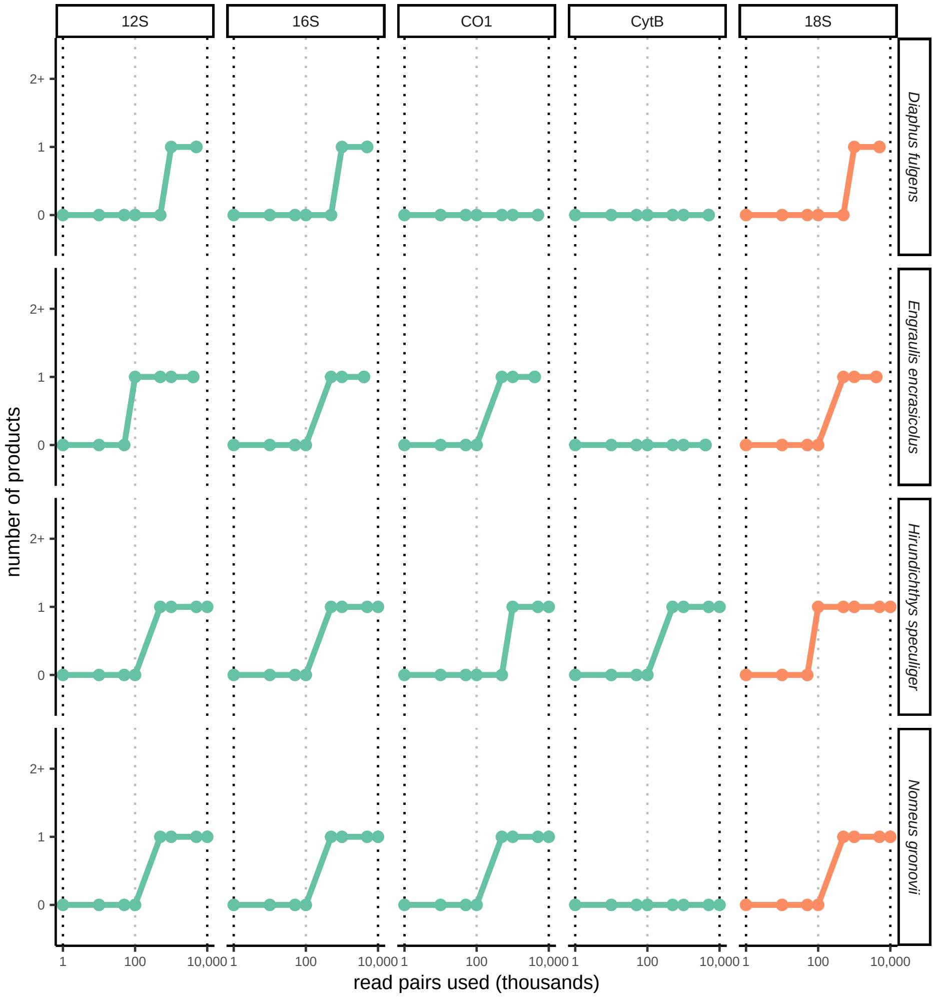

Figure S4: sPCR on the preconfigured primer panel for teleost fish. teal = mitochondrial loci, orange = nuclear ribosomal

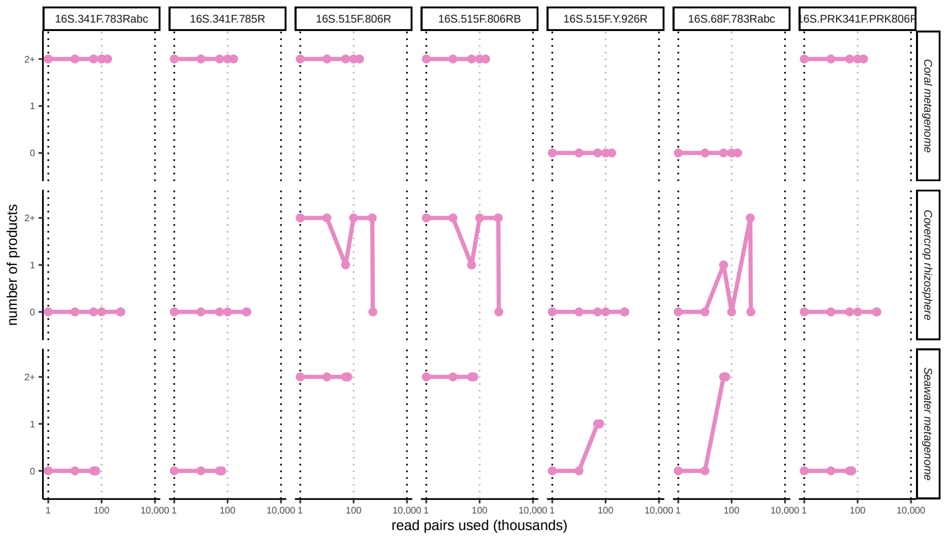

Figure S5: sPCR on the preconfigured primer panel for Bacteria. pink = ribosomal loci

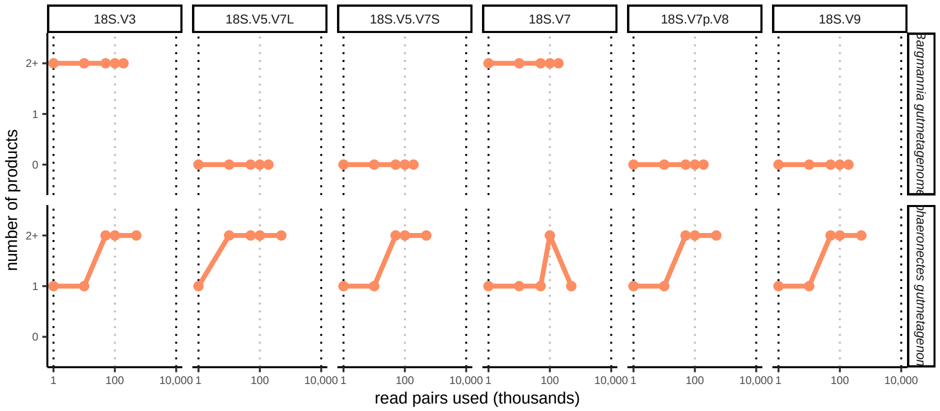

Figure S6: sPCR on the preconfigured primer panel for Metazoa. orange = nuclear ribosomal loci

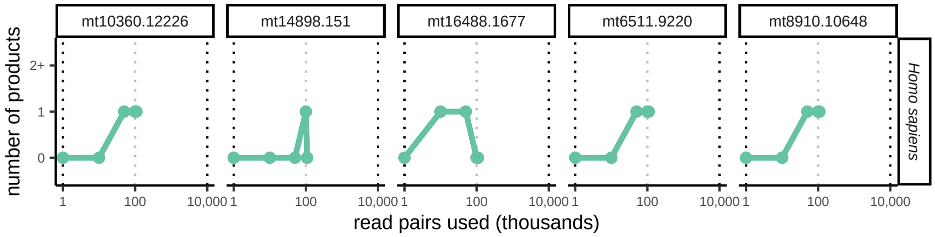

Figure S7: sPCR on the preconfigured primer panel for human mitochondrial markers. teal = mitochondrial loci.
